# Occupational Pesticide Exposures and Respiratory Health

**DOI:** 10.3390/ijerph10126442

**Published:** 2013-11-28

**Authors:** Ming Ye, Jeremy Beach, Jonathan W. Martin, Ambikaipakan Senthilselvan

**Affiliations:** 1 School of Public Health, University of Alberta, 3-276 Edmonton Heath Clinic Academy, 11405-87 Avenue, Edmonton, AB T6G 1C9, Canada; E-Mail: mye@ualberta.ca; 2 Division of Preventive Medicine, Department of Medicine, University of Alberta, 5-30 University Terrace, 8303-112 Street, Edmonton, AB T6G 2T4, Canada; E-Mail: jeremy.beach@ualberta.ca; 3 Division of Analytical and Environmental Toxicology, Department of Laboratory Medicine and Pathology, University of Alberta, 10-102C Clinical Sciences Building, Edmonton, AB T6G 2G3, Canada; E-Mail: jon.martin@ualberta.ca

**Keywords:** asthma, COPD, lung function, occupational, pesticide exposures, respiratory health

## Abstract

Pesticides have been widely used to control pest and pest-related diseases in agriculture, fishery, forestry and the food industry. In this review, we identify a number of respiratory symptoms and diseases that have been associated with occupational pesticide exposures. Impaired lung function has also been observed among people occupationally exposed to pesticides. There was strong evidence for an association between occupational pesticide exposure and asthma, especially in agricultural occupations. In addition, we found suggestive evidence for a link between occupational pesticide exposure and chronic bronchitis or COPD. There was inconclusive evidence for the association between occupational pesticide exposure and lung cancer. Better control of pesticide uses and enforcement of safety behaviors, such as using personal protection equipment (PPE) in the workplace, are critical for reducing the risk of developing pesticide-related symptoms and diseases. Educational training programs focusing on basic safety precautions and proper uses of personal protection equipment (PPE) are possible interventions that could be used to control the respiratory diseases associated with pesticide exposure in occupational setting.

## 1. Introduction

Pesticides, including herbicides, insecticides, fungicides, bactericides and rodenticides, are widely used to control pests and pest-induced diseases [1]. Worldwide, approximately five billion pounds of pesticide are consumed annually [2], among which organophosphate (OP) and carbamate insecticides (34%), dithiocarbamate fungicides (18%) and phenoxyl herbicides (12%) are the most commonly used [3]. In many occupational settings, including agriculture, fishery, forestry and food industry, pesticides have been widely used in large quantities [2]. Occupational exposures to pesticides occur during the production, transportation, preparation and application of pesticides in the workplace [1,4]. It is also quite common for agricultural workers to experience pesticide exposures even when performing tasks not specifically related to pesticide use [5,6,7]. The toxic properties of pesticides pose a potential hazard to human health. It has been estimated that the incidence rate of pesticide-related illness in the workplace was approximately 1.17 per 100,000 full time equivalent workers (FTEs) [8]. Respiratory symptoms, such as coughing, wheezing and airway inflammation, are commonly observed among people exposed to pesticides [9,10]. Epidemiological studies have attempted to investigate the association between occupational pesticide exposures and chronic respiratory diseases, such asthma, chronic obstructive pulmonary disease (COPD) and lung cancer [10,11,12].

In this review article, we critically reviewed the evidence available to date about the relationships between occupational pesticide exposures and respiratory health, including lung function, respiratory symptoms and diseases. We also reviewed a number of studies suggesting effective approaches to prevent and control pesticide-related respiratory diseases in the workplace. To review the literature, we searched English-language studies, reports and abstracts between 1990 and September 2013 in MEDLINE using key words (including synonyms and plural forms) and combinations of key words, including occupation, workplace, pesticide, insecticide, herbicide, respiratory, pulmonary, airway, lung function, infection, asthma, bronchitis, chronic obstructive pulmonary disease (COPD), and lung cancer, Searching strategy also included cross-referencing of research and review papers. Studies of non-occupational or environmental pesticide exposures were excluded.

## 2. Pesticides

### 2.1. Pesticide Classification

Pesticides are widely used in occupational settings and residential areas to prevent and control pests and pests-induced diseases [1]. Based on the target, pesticides are mainly grouped into herbicides, insecticides, fungicides, bactericides, and rodenticides (Table 1). Based on chemical properties, pesticides can also be categorized into organochlorines (OC), organophosphates (OP), carbamates, dithiocarbamates, pyrethroids, phenoxyl, triazine, amide, and coumadin compounds (Table 1). Other substances such as sulfur fumigants, urea derivatives and even botanic and biological products have also been used as pesticides in human history. For example, the element sulfur was used as a fumigant by Chinese farmers around 1000 B.C. [13]. There are historical reports of using the seed of *strychnos nuxvomica* as a rodenticide, and the root of *Derris Eliptica* (a source of rotenone) as an insecticide [13]. In addition, protein product expressed by microbe *Bacillus thuringiensis* has been used as an insecticide [14].

**Table 1:** Categories of commonly used pesticides.

Type of Pesticide	Chemicals
**Herbicide**	Chlorophenoxyl (2,4-D, 2,4,5-T and MCPA), urea derivatives, triazines (atrazine), amide (propanil), bipyridils (paraquat and diquat), glyphosate
**Insecticide**	Organochlorines [dichlorodiphenylethanes (DDT, DDD, dicofol), chlorinated cyclohexanes and benzenes (lindane, HCB), cyclodienes (aldrin, endosulfan, chlordane and toxaphene) and chlordecone (mirex), organophosphates (chlorpyrifos, diazinon, parathion, malathion), carbamates (aldicarb, aminocarb), pyrethroids (pyrethrins, permethrin, deltamethrin, cypermethrin), rotenone, *Bacillus thuringiensis* (protein product)
**Fungicide**	Dithiocarbamate, captan, captofol, pentachlorophenol, iprodione, sulphur
**Bactericide**	Triazine-S-triones, chlorine-releasing agents, chlorine, dichloronitrobenzene
**Rodenticide**	Coumadin and derivatives, anticoagulants, strychnine, sodium fluoroacetate
**Fumigant**	Methyl bromide, aluminum/zinc phosphide, sulfur

Pesticides can also be classified based on their mechanism of action. For example, OC, OP and pyrethroid insecticides are designed as neurotoxins. Phenoxyl herbicides are plant hormone analogues. Some pesticides are disruptors of normal metabolism and physiological processes, such as triazine and urea herbicides [15,16]. Rodenticide coumadin and its derivatives have the potential to depress vitamin K synthesis and thus have anticoagulant property. Sodium fluoacetate, another rodenticide, is thought to interfere with the citric acid cycle [17]. There are also pesticides, such as dithiocarbamate fungicides and amide herbicides, which are disruptors of energy production and inducers of oxidative stress [18].

The acute toxidromes of pesticides in humans are mainly due to pesticide neurotoxicity, including interference with neural conduction by targeting voltage-gated ion channels or Na+/K+ ATPase, interference with neural transmission by inhibiting acetylcholine esterase, stimulating respiratory sensory neurons or initiating pro-inflammatory signals [13]. At high dose exposures, OC, OP and pyrethroids can affect both the central neural system (CNS) and peripheral neural system (PNS) in mammals [18]. As shown in Table 2, based on their neurotoxic effects in mammals [13], pesticides can be classified into three groups.

**Table 2:** Classification of pesticides based on neurotoxicity.

Neural conduction interferer	
Organochlorine	dichlorodiphenylethanes (DDT, DDE)
	cyclodienes (aldrin, α-chlordane, γ-chlordane*, cis*-nonachlor, *trans*-nonachlor, oxychlordane, toxaphene parlar 26, toxaphene parlar 50)
	hexachlorocyclohexanes (hexachlorobenzene, b-hexachloro-cyclohexane, γ-hexachlorocyclohexane)
	chlordecone (Mirex)
Pyrethroid	pyrethrin, tetramethrin
**Acetylcholine esterase inhibitor**	
Organophosphate	parathion, malathion, methyl parathion, chlorpyrifos, diazinon,
Carbamate	aldicarb, carbofuran, carbaryl, ethienocarb, fenobucarb
**Pro-inflammatory stimulator**	
Chlorophenoxy herbicides	2,4-D (2,4-dichlorophenoxyacetic Acid)

### 2.2. Pesticide Exposures

#### 2.2.1. Overview of Occupational Pesticides Exposures

Occupational exposure to pesticides takes place during the production, transportation, preparation and application of pesticides in the workplace [1,4]. Factors involved in occupational pesticide exposures usually include application intensity, frequency, duration and method, safety behaviors (e.g., use of personal protective equipment), as well as the physiochemical and toxicological profiles of the pesticides in use [19]. In occupational settings, persons working directly and frequently with pesticides are groups with the highest risk of exposure [4]. Additionally, family members of pesticide applicators can have substantial exposures to pesticides [20,21]. In addition to the fact that occupational pesticide exposure is quite common among agricultural workers and their family members [5,6,7], accidental spills of pesticides, leakages, incorrect uses of equipment, and non-compliance with safety guidelines, are the leading causes of occupational pesticide exposures [4,22]. Compared to environmental exposures where levels of exposure tend to be fairly low, occupational exposures to pesticides are often at relatively high doses, whether acute or chronic [1]. 

#### 2.2.2. Routes of Pesticide Exposures

Respiratory inhalation and dermal absorption are considered as the primary routes of exposures to pesticide in occupational settings [1,23]. Respiratory exposures usually occur when applying highly volatile pesticide products, especially for those working with no respiratory protective equipment (e.g., mask with filter) or in a poorly ventilated working environment [24]. In agricultural occupations, typically about 10% of total pesticide exposure occurs via the respiratory route, with the rest through either dermal absorption or digestion [24]. For non-volatile pesticides, respiratory inhalation also occurs when pesticides are sprayed an inhalable form. Dermal absorption occurs through direct skin contact with pesticides or from clothing and tools that are contaminated with pesticide residues [10]. A study of Greek tobacco-growing farmer suggested that dermal exposure was the major route of exposure (58%) during occupational pesticide uses [25]. Dermal exposure and ingestion may also be relevant for systematic inflammation or sensitization after high level exposures to pesticide at the workplace [23]. 

The physiochemical properties of the particular pesticide, temperature, humidity, weather conditions, personal hygiene (e.g., hand washing), and use of personal protective equipment are all factors associated with pesticide exposures [26,27]. For example, organophosphate and carbamate insecticides can be efficiently absorbed by the skin due to their high lipid solubility [28]. Certain organochlorine insecticides, such as DDT (dichlorodiphenyltrichloroethane), lindane, aldrin and chlordane, are more lipid soluble than others and thereby more efficiently absorbed by the skin [28]. In contrast, due to the low lipid solubility, pyrethroid insecticides are poorly absorbed though intact skin, but can be efficiently absorbed through inhalation and ingestion [28]. Chlorophenoxyl herbicides are often in a form of salts, which results in a low volatility and lipid solubility and makes phenoxyl compounds well absorbed by the gastrointestinal tract following ingestion, but less well absorbed by the lungs, and least well by the skin [28]. Methyl bromide, a halogenated fumigant, exists as a colorless and odorless volatile liquid and thus has poor olfactory warning properties, which increase the likelihood of exposure through inhalation [29]. Respiratory exposure also occurs through airway inhalation of pesticide-contaminated aerosols or particulate matters (PM) [30]. The hygroscopicity and mass-mediated aerodynamic diameter (MMAD) of pesticide-containing particles are important in determining their local deposition in airways, and hence potentially the site of toxicity [31]. 

#### 2.2.3. Biomonitoring of Pesticide Levels

Due to variations in exposure magnitude and duration, routes of absorption (skin, respiratory tract, gastrointestinal tract), and physiological variability between exposed individuals, it is often difficult to quantitatively assess the effective dose of a pesticide an individual has received either by measuring working hours or by monitoring the contamination level of the workplace. Biomonitoring of pesticide levels in biospecimen or using biomarkers has been considered as an alternative approach to assess pesticide exposures. It can provide an objective measure of the physiological burden of a pesticide on the human body. For example, using the biomonitoring data, the US National Health And Nutrition Examination Survey (NHANES) has been able to identify that the majority of the US population had OP metabolites detectable in their urine samples [32]. It has also been shown that reduced levels of red blood cell (RBC) cholinesterase were significantly associated with the number of years of using OP pesticides after adjusting for age [33]. 

In the US Farm Family Exposure Study, researchers were able to assess pesticide exposure profiles by measuring urinary levels of herbicides 2,4-D and atrazine, and TCP (3,5,6-trichloro-2-pyridinol), a metabolite of organophosphate insecticide chlorpyrifos [34,35,36]. As part of the Farm Family Exposure Study, Curwin *et al*. also found a significant correlation between urinary levels of herbicide atrazine and the atrazine exposure levels measured by hand wipe samples [36]. Nevertheless, in a study of pesticide with relatively short half-life in the body, Perry *et al.* showed poor agreement between self-reported exposure and urinary measures of deethylatrazine (a major atrazine metabolite) level [37]. 

Although biomonitoring approach has been accepted as an alternative to estimate exposures, a biomonitoring method often requires complex and costly sample collection, transportation, and analytical methods, sometime involving invasive procedures such as blood sample collections, which may make it less acceptable to participants [38,39,40,41,42]. 

## 3. Pesticide-Related Respiratory Symptoms and Diseases

Due to their inherent biological reactivity, pesticides are potentially hazardous to human health. Globally, around 300,000 deaths per year are resulted from acute pesticide poisoning, with organophosphates, organochlorines and aluminium phosphide being reported most frequently as the cause [43]. According to the US Sentinel Event Notification System for Occupational Risks (SENSOR)-pesticides surveillance program, the overall incidence rate of acute occupational pesticide-related illness was 1.17 per 100,000 full time equivalent workers (FTEs) and insecticides were responsible for 49% of all illnesses [8]. Moreover, the incidence rate among agricultural occupations, where pesticides are extensively and intensively used, was much higher (18.2/100,000 FTEs) compared to those employed in non-agricultural occupations (0.53/100,000 FTEs) [8]. A summary of the adverse health effects of environmental chemicals suggests that pesticide exposures may cause asthma (both new incidence and exacerbation of preexisting disease), chronic obstructive pulmonary disease and even lung cancer [10].

### 3.1. Respiratory Symptoms

Respiratory symptoms that have been reported in association with pesticide exposures include wheezing, airway irritation, dry/sore throat, cough, breathlessness and chest tightness. A cross-sectional study of workers in a bottling plant showed that in comparison with controls, pesticide processing workers had a significantly higher risk of developing respiratory symptoms, including chronic cough in females (OR = 1.29, 95% CI: 1.15–15.84), dyspnea grades 3 and 4 (OR = 1.11, 95% CI: 1.06–1.97 in females; OR = 2.35, 95% CI: 1.50–4.10 in males), throat irritation in males (OR = 1.36, 95% CI: 1.10–3.50), nasal catarrh (OR = 2.08, 95% CI: 1.12–3.40 in females; OR = 2.15, 95% CI: 1.15–4.10 in males), and nasal dryness (OR = 1.15, 95% CI: 1.05–2.91 in females; OR = 1.19, 95% CI: 1.10–3.15 in males) [44]. In addition, acute respiratory symptoms, such as cough, wheezing, chest tightness, dyspnea, throat irritation and dryness, nose secretion and dryness, were significantly increased across the work-shift among pesticide workers [44]. 

A number of other studies have also reported an excess of respiratory symptoms among farm workers exposed to pesticides. A study of livestock farm workers from Iowa reported that after adjusting for age and smoking, respiratory symptoms including phlegm (OR = 1.91, 95% CI: 1.02–3.57), wheezing (OR = 3.92, 95% CI: 1.76–8.72) and flu-like symptoms (OR = 2.93, 95% CI: 1.69–5.12) were significantly associated with pesticide use, although the results may have been affected by concurrent exposures to other environmental agents such as ammonia and animal antigens [45]. In spite of non-significant results, Hashemi *et al*. in their study of work-related symptoms among Iranian farmers also reported that pesticide use was associated with an increased risk of wheezing and phlegm [46]. A study of the Farm Family Health and Hazard Surveillance Program (FFHHSP) among Ohio grain farmers in the US showed that personal involvement with pesticides was associated with a high prevalence of chronic cough [47].

In addition to these results suggesting the overall respiratory effect of unspecified pesticides, there have been some studies focusing on particular types of agent. For example, the neurological effects caused by cholinesterase inhibiting pesticides, such as OPs and carbamates, have been recognized to affect lungs and airways, leading to respiratory symptoms, impaired lung function and respiratory diseases. A matched case-control study of agricultural workers in Eastern India showed that compared to controls, agricultural workers who sprayed organophosphate and carbamate pesticides had significant depletion of red blood cell acetylcholinesterase (AchE), and the depletion of AchE was significantly associated with almost all respiratory symptoms, including runny or stuffy nose (OR = 2.85, 95% CI: 1.98–4.63), sore throat (OR = 1.76, 95% CI: 1.29–2.43), dry cough (OR = 2.83, 95% CI: 1.92–4.41), wheezing (OR = 1.78, 95% CI: 1.33–2.46), breathlessness (OR = 2.41, 95% CI: 2.06–3.82), chest tightness (OR = 3.26, 95% CI: 2.23–5.17) and dyspnea (OR = 2.63, 95% CI: 1.89–4.13), as well as chronic bronchitis (OR = 2.54, 95% CI: 1.48–3.74) and doctor diagnosed asthma (OR = 1.34, 95% CI: 1.09–1.79) [48]. Hoppin *et al.* reporting results from the Agricultural Health Study (AHS) found that exposures to the OP pesticides dichlorvos and phorate in the past year were significantly associated with wheezing among commercial pesticide applicators (OR = 2.48, 95% CI: 1.08–5.66 and OR = 2.35, 95% CI: 1.36–4.06, respectively) after adjusting for age, BMI, smoking, asthma/atopy and previous use of pesticide [49]. Hoppin *et al*. also demonstrated a dose-response trend (*p*-value for trend < 0.01) for the association between organophosphate pesticide chlorpyrifos and wheezing [49]. In a study of Kenyan agricultural workers, Ohayo-Mitoko *et al*. reported that acetylcholinesterase inhibiting pesticides, including dimethoate, malathion, benomyi, mancozeb, methomyi, aldicarb, and propineb, were associated with a higher prevalence of respiratory symptoms, such as chest pain, cough, running nose, wheezing, difficulties in breathing, shortness of breath, and irritation of the throat [50]. Ciesielski *et al.* also found that pesticide exposure related cholinesterase inhibition was associated with chest pain and difficulty in breathing in a study of North Carolina migrant farmworkers [51]. However, neither study identified a significant dose-response effect of cholinesterase level on respiratory symptoms [50,51].

Exposures to other types of pesticides, such as pyrethroid insecticides, certain herbicides and fumigants, can also lead to respiratory symptoms. Hoppin *et al*. in their study of farmers in the Agricultural Health Study (AHS) showed that herbicides alachlor (OR = 1.23, 95% CI: 1.06–1.41), atrazine (OR = 1.18, 95% CI: 1.05–1.32), S-ethyl-dipropylthiocarbamate (EPTC) (OR = 1.37, 95% CI: 1.08–1.73), petroleum oil (OR = 1.26, 95% CI: 1.09–1.47) and trifluralin (OR = 1.15, 95% CI: 1.02–1.30), and the insecticide permethrin (OR = 1.28, 95% CI: 1.06–1.55), were significantly associated with wheezing [49]. It has also been reported that office workers in California experienced shortness of breath and irritation of the respiratory tract after accidentally inhaling cypermethrin, a pyrethroid insecticide [52]. A case study in Tanzania reported that inhabitants of houses sprayed with lambda-cyhalothrinnose, a pyrethroid insecticide, had nose or throat irritation accompanied by sneezing or coughing [53]. Although no dose-response relationship was found, in a study of Indonesian farmers, Kishi *et al*. showed that the respiratory symptoms, including dry throat, sore throat, difficulty in breathing and chest pain, were significantly associated with the pesticide spray season [54]. Moreover, a study of farmers in Nepal suggested that activities regarding to insecticide or fungicide uses, including the spraying duration and mixing pesticides were significant predictors of throat discomfort and respiratory depression [55]. 

Due to its neurotoxicity, occupational exposures (poisoning) to fumigant methyl bromide can also cause respiratory symptoms, including respiratory irritation, respiratory distress (shortness of breath), coughing and pulmonary injury (edema) [29,56,57,58]. These respiratory symptoms often accompany other local or systematic symptoms including dizziness, vomiting, fatigue, headache, abdominal pain, tremor, seizures, ataxia paresthesia and dysfunction of other organs [57,59,60]. 

In summary, a large number of studies have identified associations between respiratory symptoms and pesticide exposure, but to date the findings have been relatively non-specific both in terms of the agents causing the risk and the symptoms caused, which makes interpretation of the data complex. Despite this, there does seem to be good evidence that at least some pesticides cause acute and chronic respiratory symptoms.

### 3.2. Lung Function

A number of papers have suggested that the use of pesticides in occupational settings is associated with impaired lung function. For example, in a patient reported as a case of occupational asthma related to chronic exposure to the fungicide captofol showed a substantial and persistent decrease in FEV_1_ (forced expired volume in 1 s) [61]. A cross-sectional study among the pesticide-processing workers showed that there was significant reduction in FVC (forced vital capacity), FEV_1_ and FEF_25%–75%_ (the forced expiratory flow between 25% and 75% of forced vital capacity) in comparison to controls [44]. Another cross-sectional study of 102 pesticide sprayers and 69 non-sprayers in state farms of Ethiopia showed that pesticide sprayers in the age group of 15–24 years had significantly reduced FEV_1_ and FVC compared to controls [62]. A similar study conducted among agricultural pesticide sprayers in Spain suggested that short term exposure to pesticides was related to reduction in FEV_1_, while long term pesticide exposure was associated with reduction in FEF_25%–75%_ after adjusting for age, gender, smoking status, body mass index (BMI), height, alcohol consumption, paraoxonase 1 (PON1) polymorphism and cholinesterase levels [63]. Moreover, in a study of farm workers in Sri Lanka, Peiris-John *et al*. suggested that the OP insecticide related decrease in FEV_1_ and FVC occurred among those in agricultural occupations [64]. When comparing the respiratory function of pesticide factory workers with controls in Lebanon, Salameh *et al*. also found a significant reduction in FEV_1_, FEF_25%-75%_, and FEV_1_/FVC ratio [65]. In addition, in a study conducted among agricultural workers in Colorado and Nebraska, an interaction between pesticide and endotoxin was identified with those workers reporting both exposures having a significantly greater endotoxin-related reduction in FEV_1_ [66]. 

Impaired lung function was also associated with organophosphate or carbamate insecticide induced cholinesterase inhibition. In a matched case-control study of agricultural workers in India, exposures to organophosphate and carbamate insecticides were significantly associated with reductions in FVC, FEV_1_, FEV_1_/FVC ratio, FEF_25%-75%_ and peak expiratory flow rate (PEFR), which was also significantly correlated with cholinesterase inhibition [48]. In addition, a cross-sectional study of pesticide sprayers in Indian mango orchards similarly showed a correlation between impaired lung function and reduction of acetylcholinesterase and butylcholinesterase activities [67]. 

Other than the adverse effect on dynamic lung volumes, occupational exposures to pesticides may also lead to impairment of gas exchange in the lung. Two studies among farmers in Costa Rican and Western Cape showed a relationship between long-term low level paraquat exposures and exercise-associated oxygen desaturation, suggesting paraquat may cause gas exchange abnormalities [68,69]. 

Both obstructive and restrictive abnormalities have been reported in association with occupational pesticide exposures. In the Spanish study mentioned above, FEV_1_/FVC ratio was decreased among farm pesticide sprayers (although the change was not significant) [63], suggesting an obstructive abnormality. In India, long-term exposure to cholinesterase-inhibiting insecticides among agricultural workers was also associated with a significant decrease in the FEV_1_/FVC ratio [48]. In Sri Lanka, the acute seasonal low-level exposure to organophosphate pesticides among farmers was associated with a normal FEV_1_/FVC ratio but a reduction of both FVC and FEV_1_ [64], suggesting a restrictive abnormality. In a study of pesticide spraying workers in mango plantation in India, the author suggested that a restrictive type of impairment of lung function was related to the exposures to organophosphate and organochlorine insecticide [70]. Additionally, in a study of farm operator and their spouse in Colorado in US, pesticide poisoning was significantly associated with lower FVC and FEV_1_ among current smokers, again suggesting a restrictive defect [71]. 

There were also studies reporting no clear association [72,73,74] between pesticide exposure and lung function. These results may be due to the uncontrolled social and environmental factors [73,74], a “healthy worker effect” [73], better awareness of pesticide associated hazards among some workers [72], or other inherent issues with study design [72,73,74]. 

### 3.3. Occupational Asthma

In the past decade, asthma has been recognized as the most commonly reported occupational lung diseases [75], although there are variations in the attributable fraction (defined as one minus the reciprocal of relative risk) for occupational asthma in different populations [76,77,78,79]. Occupational asthma can be associated with significant medical and socioeconomic consequences [80,81,82]. 

Pesticide exposures have been associated with asthma in a number of occupational settings [83,84]. For example, in France, a case of persistent asthma was linked to acute inhalation of the organophosphate insecticide dichlorvos for 8 hours in a closed kitchen [85]. Two cases of occupational asthma were reported in UK following exposure to fungicides fluazinam and chlorothalonil [86]. In Belgium, a case of occupational asthma was linked to the chronic exposure to tetramethrin, a pyrethroid insecticide [87]. In addition, a case series of individuals with reactive airways dysfunction syndrome (RADS), a subtype of work-related irritant-induced asthma (IIA), were thought to be related to exposures to unspecified herbicide and insecticide diazinon [88]. 

In a population-based study of occupational risk factors for asthma and respiratory symptoms in Singapore, LeVan *et al*. found that vapor exposure from pesticides (unspecified) was associated with non-chronic cough or phlegm (OR = 1.14, 95% CI: 1.03–1.27), chronic dry cough (OR = 1.55, 95% CI: 1.19–2.01), and adult-onset asthma (OR = 1.34, 95% CI: 1.15–1.56) [89]. A cross-sectional study conducted among 1,379 Brazilian agricultural workers showed that pesticide (unspecified) exposure in agricultural occupations was associated with a higher prevalence of adult-onset asthma (OR = 1.54, 95% CI: 1.04–2.58) [90]. In this study, the authors also suggested that the effect of pesticide exposures on asthma was stronger in women than in men [90]. In Canada, a cross-sectional study on male farmers in Saskatchewan suggested that self-reported asthma was associated with the use of carbamate insecticides (OR = 1.8, 95% CI: 1.1–3.1) [91]. This study was the first population-based study to report an association between asthma and use of carbamate insecticides. No significant association was found between asthma and use of organophosphate insecticides in this study [91].

The Agricultural Health Study (AHS) in the US reported that adult-onset asthma was associated with exposure to pesticides, including organophosphate insecticides, carbamate insecticides and herbicides alachlor, atrazine and paraquat [11,49,92,93]. In these studies, dose-dependent relationships were also observed between asthma symptom of wheezing and application of organophosphate insecticides chlorpyrifos and parathion and herbicides atrazine and paraquat [49,92]. Hoppin *et al*. showed highly significant associations between adult-onset atopic asthma among male farmers and agricultural uses of pesticides coumaphos (OR 2.34; 95% CI: 1.49–3.70), heptachlor (OR 2.01; 95% CI: 1.30–3.11), parathion (OR 2.05; 95% CI: 1.21–3.46), 80/20 mix (carbon tetrachloride/carbon disulfide) (OR 2.15; 95% CI: 1.23–3.76) and ethylene dibromide (OR 2.07; 95% CI: 1.02–4.20) [11]. For nonatopic asthma, Hoppin *et al*. showed that DDT had the strongest association (OR 1.41; 95% CI: 1.09–1.84) among male farmers [11]. Among female farmers in the AHS, agricultural pesticide exposures, including seven insecticides (carbaryl, coumaphos, DDT, malathion, parathion, permethrin, and phorate), two herbicides (2,4-D and glyphosate) and a fungicide (metalaxyl), were more associated with atopic asthma than non-atopic asthma [93]. Hoppin *et al*. also suggested that growing up on a farm might modify the association between pesticide use and atopic asthma [93]. Consistent with the AHS study, a recent study of French farmers also suggested that pesticide exposures were more associated with allergic asthma (OR = 1.97; 95% CI: 1.43–2.73) than non-allergic asthma (OR = 1.24; 95% CI: 0.88–1.76) [94]. Given that there is little antigenicity of chemical pesticide, pesticide-induced or promoted allergic/atopic asthma may be due to the indirect effect of pesticides on the immune system, such as interfering with Th-1/Th-2 (T-helper) balance or pesticide-induced oxidative stress [23,95]. 

Pesticide use has also been associated with asthma exacerbations and health outcomes of patients with occupational asthma. In a retrospective cohort study of outdoor pesticide applicators in Australia, the mortality rate from asthma was higher (SMR = 3.45; 95% CI: 1.39–7.10) in workers who were occupationally exposed to insecticides, including the organochlorine insecticide DDT and acetylcholine esterase inhibiting insecticides carbaryl and chlorpyrifos, compared to the general population [96]. 

Non-significant association [73,97] and inverse-effect relationships [74,98] between pesticide exposure and asthma have also been observed. For example, a cross-sectional study conducted among female indigenous plantation workers in Costa Rica found a protective relationship between organophosphate pesticide exposure (terbufos and chlorpyrifos) and asthma or lung function [74]. However, in these studies, often only a small number of subjects were investigated [73,74,98] or many important social and environmental risk factors of asthma, such as household income, educational levels and growing-up in the farming environment, were not considered [73,74] when assessing the association between occupational pesticide exposures and asthma. Therefore, although weak and statistically non-significant relationships were reported [72,73,98], most epidemiological studies have suggested a significant association of occupational pesticide use with asthma [11,49,74,85,87,90,91,93,96].

### 3.4. Chronic Bronchitis and COPD

Many studies have suggested that exposure to pesticides, especially in occupational settings, is associated with chronic bronchitis and COPD. In a matched case-control study of agricultural workers in India, a higher prevalence of chronic bronchitis was associated with OP and carbamate pesticide exposures (OR = 2.54, 95% CI: 1.48–3.74) [48]. A study of pesticide producing workers in Poland showed that a higher prevalence of diagnosed COPD (19.3% *vs.* 3%; *p* = 0.002) was associated with pesticide exposures at work after adjusting for smoking status [99]. In addition, there was a negative correlation between FEV_1_/FVC index and duration of pesticide exposures in this study [99]. A case-control study in Lebanon showed a similar positive relationship between pesticide exposure and chronic bronchitis [100]. In the American Agriculture Health Study (AHS), Hoppin *et al*. showed that 11 pesticides, including organochlorine pesticides (heptachlor chlordane, DDT, lindane and, toxaphene), organophosphate pesticides (coumaphos, diazinon, dichlorvos, malathion, parathion) carbamate pesticides (carbaryl and carbofuran), permethrin, chlorophenoxy herbicides (2,4,5-T and 2,4,5-TP) and two other herbicides (chlorimuron-ethyl and petroleum oil) were significantly associated with chronic bronchitis [12]. In addition, in the AHS study, farmers with a history of high exposures to pesticide had a higher prevalence of chronic bronchitis (OR = 1.85, 95% CI: 1.51–2.25) [12]. Another study using the same dataset found that the incidence of chronic bronchitis among female non-smoking farmers was significantly related to the application of five pesticides, including insecticides dichlorvos (OR = 1.63, 95% CI: 1.01–2.61) and DDT (OR = 1.67, 95% CI: 1.13–2.47), and herbicides cyanazine (OR = 1.88, 95% CI: 1.00–3.54), methyl bromide (OR = 1.82, 95% CI: 1.02–3.24) and paraquat (OR = 1.91, 95% CI: 1.02–3.55) [101]. A recent cross-sectional study of AGRIculture and CANcer (AGRICAN), a French agricultural cohort, showed that risk of chronic bronchitis among farmers was significantly associated with pesticide poisoning (OR = 1.67, 95% CI: 1.08–2.58 for those without healthcare; OR = 1.64, 95% CI: 1.11–2.41 for those with healthcare), but not significantly with activities of using pesticides [102], suggesting that episodes of high-dose acute pesticide poisoning possibly contribute more to COPD than long term low level exposures. 

### 3.5. Lung Cancer

Occupational pesticide exposure has been linked to lung cancer, especially in agricultural settings. For example, a study of pest control workers in Florida suggested that longer duration exposure to organophosphate and carbamate insecticides and phenyoxyacetic acid herbicides was associated with a higher mortality rate of lung cancer (OR = 1.4, 95% CI: 0.7–3.0 for subjects licensed 10–19 years; OR = 2.1, 95% CI: 0.8–5.5 for those licensed 20 years or more) [103]. In a nested case-control study arising from the Agriculture Health Study (AHS), after controlling for age and tobacco smoking, Alavanja *et al*. showed statistically significant dose-response relationships between pesticide exposures, including insecticides chlorpyrifos and diazinon and herbicides metolachlor and pendimethalin, and risk of lung cancer [104]. Similar results were replicated in later studies of the AHS cohort for chlorpyrifos [105], diazinon [106], metolachlor [107] and pendimethlin [108]. The organochlorine insecticide dieldrin and the carbamate insecticide carbofuran were also reported to be positively associated with the risk of lung cancer [109,110]. Samanic *et al*. showed that the highest tertile of lifetime exposure to herbicide dicamba was significantly associated with an increased risk of lung cancer (RR = 2.16, *p*-value for trend = 0.02) [111]. Moreover, arsenical pesticides, which are not currently used, were linked to lung cancer [112,113,114]. However, there were also a number of studies showing non-significant associations [115,116,117,118,119,120,121,122,123] or negative relationships [124,125,126,127,128,129,130,131] between occupational pesticide use and lung cancer. 

Although a number of studies have controlled for age and smoking status when assessing the association between pesticide exposure and lung cancer, some important risk factors of lung cancer, such as indoor/outdoor air pollutants [132,133], life styles and psychosocial factors [134] and genetic predisposition [135], have not been routinely taken into account. In addition, the impurities or promoting agents in pesticide formulae, such as dioxin and dioxin-like contaminants of phenoxy herbicides (2,4-D, 2,4,5-T and MCPA) [136,137], might have contributed to the significant association found between some pesticide exposures and lung cancer [103,136,137]. Therefore, current studies did not provide conclusive evidence connecting occupational pesticide exposure with lung cancer. 

### 3.6. Other Respiratory Diseases

In addition to asthma, COPD and lung cancer, other respiratory diseases have also been linked to occupational pesticide exposures. For example, in the analysis of A Case Control Etiologic Study of Sarcoidosis (ACCESS) data, Newman *et al*. found that occupational exposure to insecticides was associated with an increased risk of sarcoidosis [138]. Slager *et al*., using data from the Agricultural Health Study, found that herbicides 2,4-D, glyphosate and petroleum oil (an additive in the herbicide formula to increase the phytotoxocity), the insecticide diazinon and the fungicide benomyl were positively associated with current rhinitis [139,140]. In a study of grape farmers in Greece, exposures to paraquat and other bipyridyl herbicides increased the risk of developing allergic rhinitis [141]. 

In a cross-sectional study of farm residents in northeastern Colorado, experience of pesticide poisoning was significantly associated with a number of respiratory problems including cough, allergy, wheeze, and organic dust toxic syndrome (ODTS) among non-smokers [71]. Although no significant association was found between pesticide exposures and farmer’s lung, Hoppin *et al.* suggested that pesticide exposures, especially to organochlorine and carbamate pesticides, along with the causative exposure to thermophilic fungi from farming activities, such as silage handling and animal exposures, may collectively contribute to the incidence of farmer’s lung [142]. In addition, although respiratory infection has been linked to exposure to organochlorine pesticides in young children [143,144], there is a lack of evidence clearly demonstrating such a link between pesticide exposures and respiratory tract infection in occupational settings.

## 4. Prevention of Pesticide Exposures and Related Respiratory Diseases

### 4.1. Regulation of Pesticide Uses

According to the US Environmental Protection Agency (EPA), approximately five billion pounds of pesticides are used annually worldwide [2]. A cross-sectional study of pesticide handling practices among Cambodian farmers suggested that most of the pesticides used in agriculture belonged to the World Health Organization (WHO) class I (extremely and highly dangerous) and class II (moderately dangerous) categories [145]. Therefore, one immediate aim might be to reduce the use of WHO Classes I and II pesticides, and replace these pesticides with less-toxic alternatives, especially in developing countries [146]. In Europe and North America, government agencies, such as the EPA in the US, the PMRA (Pest Management and Regulation Agency) in Canada, and the EEA (European Environment Agency) in the European Union, control pesticide use quite closely. These government agencies put forth policies and regulations on production and use of pesticides. Governmental regulations for reducing the use of pesticides include mandate reduction through national policies and enforcement [147,148], restricted registration [149] and taxation and attrition [150] on old toxic pesticides. For example, in Canada, there have been substantial decreases in cosmetic use of pesticide (from 25% to 11%) and hiring of lawn care companies (from 15% to 5%) after a province-wide ban of cosmetic pesticides in 2009 in Ontario [151]. 

Osteen *et al*. suggested that societal values towards risks and benefits of pesticide uses could profoundly affect pesticide policy and public preferences for reducing pesticide exposure so as to maximize the efficiency of pesticide regulation and policy implementation [152]. Hernke *et al*. also pointed out that scientific uncertainties and gaps in pesticide toxicology may hinder the generation of new policy on restricting pesticide uses [153]. In addition, political priority in sustainable development and efficient social connections between partnerships are critical for successful implementation of new pesticide policy aiming to reducing pesticide uses and pesticide-related risk [153].

### 4.2. Enforcing Safety Behaviors in Workplace

A study of pesticide exposures among farmworkers suggests that workplace safety behaviors are critical for preventing occupational pesticide exposures [154]. Safety behaviors in workplaces include wearing personal protective equipment (PPE), showering after work, wearing and changing clean clothes between work shifts and frequently washing hands at work [154]. 

Wearing personal protective equipment (PPE), such as a respirator, goggles and protective over-clothes, is an effective way to reduce risk of developing pesticide-induced respiratory diseases when handling pesticides. For example, a study of Cambodian farmers showed that the risk of acute pesticide poisoning was reduced by 55% among more highly educated farmers who adopted extra personal protective measures (OR = 0.45, 95% CI: 0.22–0.91), suggesting a practical approach to improve safe pesticide management [145]. Gomes *et al.* showed that AChE activity was found to be negatively associated with the use of gloves, work coveralls, and other PPE [27]. Bradman *et al*. also showed the importance of wearing protective gloves in reducing pesticide exposures among strawberry harvesters [5]. A study of farmer perceptions on pesticide use practices in Ghana also showed that pesticide poisoning occurred more often among farmers who generally did not wear protective clothing [155]. In addition, a study of farmers who used pesticides, including dithiocarbamates, pyrethroids and organophosphates in rural Indonesia, showed that those who wore no mask/respirator, wet clothing or short-sleeves, had greater skin contact with pesticides, and those who smoked during spraying were at the greatest risk for developing health problems [156]. In a study of the tobacco-growing farmers in Malaysia, Nordi *et al*. found that refraining from smoking while spraying, using a well maintained sprayer, wearing a hat while spraying, and changing clothes immediately after spraying, significantly reduced the likelihood of acute pesticide-related symptoms, especially among male farmers [157]. 

Unfortunately, awareness and knowledge of risk of pesticide exposures do not always guarantee the safety behaviors when handling pesticides. A study of tobacco farmers in northern Greece indicated that most farmers had good knowledge of potential hazards caused by pesticides [25]. However, a significant proportion of farmers (46%) reported not using any personal protective equipment and less than 10% of farmers reported using comprehensive personal protection equipment, including a face mask, gloves and coveralls on a regular basis when spraying pesticides [25]. In a cross-sectional survey of 1,102 farmers in Australia, up to 40% of farmers, did not use PPE routinely when handling pesticides [158]. This study also suggested that younger age and farm chemical training was strongly associated with PPE use [158]. A similar study among California farmers also suggested that farmers of younger age, male sex and more concerned about specific health problems were much more likely to use personal protective equipment [159]. 

Several studies have suggested a low level of knowledge and awareness of pesticide toxicity and potential health hazards among farmers in developing countries [160,161,162]. Despite clear evidence connecting pesticide exposure levels with job tasks, there has been little study of the effect of safety training on pesticide exposures [154]. An educational program in South India successfully improved knowledge, awareness and practice of safety measures of pesticide uses among agricultural workers [163]. A randomized controlled intervention among Wisconsin dairy farmers showed that a six-month educational intervention program had significant effects on increasing use of personal protective equipment, especially gloves and other protective gears, including footwear, apron, protective eyewear and approved respirator, during pesticide handling [164]. 

In summary, continuous education and training programs on the potential health risk of pesticide exposures, with an emphasis on basic safety precautions and uses of personal protection equipment (PPE), and enforcement of safety behaviors and measures during pesticide handling, all appear useful for preventing pesticide exposures and related respiratory diseases. 

### 4.3. Integrated Pest Management Strategy

Integrated Pest Management (IPM) is a strategy integrating diverse methods and practices to achieve effective and economic pest controls. According to the US Department of Agriculture, in an Integrated Pest Management (IPM) program, pesticide will be “applied as a last resort in suppression systems” and “selected based on least negative effects on environment and human health” [165]. 

An IPM strategy has the potential to reduce unnecessary pesticide applications. For example, other than wearing personal protection equipment, using alternative pesticides with lower volatility and lower concentrations of active ingredients can significantly reduce occupational pesticide exposures, especially the exposure related with respiratory outcomes [24]. There has been great reduction in use of organophosphate insecticides after substituting them with pyrethroids [166,167]. In addition, genetically modified (GM) crops, as well as those naturally-bred crops with pest resistance, have the potential to reduce the use of pesticides [168,169,170]. Governmental actions through regulations and policies, another approach of the IPM [171], are also effective in reducing pesticide exposures.

Advocacy and educational programs have been proved as an effective approach in the IPM to reduce the use of pesticides. A study in India suggested that educating farmers with knowledge of the IPM and the subsequent practice of IPM by farmers significantly reduced the use of pesticides, particularly organophosphate insecticides, which in turn was associated with a 50% reduction in the incidence of acute pesticide poisoning [172]. In addition, an integrated, community-based health promotion program has been shown to be effective in reducing the pesticide-related risk on small farms [173]. 

## 5. Potential Issues for Consideration In Future Research

### 5.1. Pesticide Exposures and Doses

In epidemiological studies, accurate exposure assessment is crucial for identifying adverse health effects. In a review paper on pesticide exposures among farmworkers, Hoppin *et al*. suggested three approaches to measure pesticide exposures in current studies: (1) personal measurements; (2) scenario-based assessment; and (3) biomonitoring measurement [19]. 

Personal measurements, such as hand-wipe samples or samples from masks or respirators, measure pesticide concentration at the immediate contact interface between subjects and pesticides, but do not measure the actual pesticide doses into the human body [19]. In a scenario-based approach, pesticide exposures are often measured or modeled by questionnaire-based measurements or job titles, both liable to error and bias [174]. 

Using biomarkers or biomonitoring levels of pesticides or their metabolites is an objective measurement of pesticide exposures, and is also considered as an approach to measure doses or actual body burden arising from pesticide exposures [175]. However, the use of biomonitoring approach has limitations, especially when short time windows are required for accurately capturing the peak level of pesticide exposures. Bio-markers may be good for organochlorine (OC) pesticides, since they typically have a long half-life, and so serum levels can be used as a marker of past or cumulative OC exposure. However, for non-persistent pesticides, such as OPs, biomarkers have limitations if trying to estimate cumulative exposure and thus often studies of chronic exposure mainly rely on questionnaire-based approaches [176]. Moreover, in biological measurement of non-persistent pesticides, the sampling time frame and temporal variability are critical for the validity of data analyses and result interpretation [175]. For example, in measuring sub-chronicle OP exposures, the AChE level in blood or erythrocytes can be used as a biomarker, since this effect can last for 3–4 months [176], although it is considered relatively insensitive and prone to error. Nevertheless, it cannot be used to evaluate carbamate exposure, as AchE inhibition by carbamates lasts only a few minutes [176]. 

Biomonitoring measures have additional benefit of integrating pesticide exposures from all physiological pathways [175]. On occasions, multiple biomarkers can be used for assessing single exposures to pesticides. Urinary metabolites of pesticides such as dialkyl phosphates and TCP (3,5,6-trichloro-2-pyridinol, a metabolite of chlorpyrifos), and plasma butyl cholinesterase (BuChE) and erythrocyte acetyl cholinesterase (AChE) activities, can all be used as biomarkers for measuring OP exposures [177]. Studies of these three biomarkers have shown an inverse relationship between the acute concentration of urinary TCP and the sub-chronic activities of plasma BuChE and/or erythrocyte AChE when assessing chlorpyrifos exposures [178,179]. 

Indirect exposures from “take-home” pesticides or overspray of residential areas as a consequence of occupational exposures may also have effect on human respiratory health [180,181,182]. For example, on the farm, pesticide applicators or sprayers often have substantial exposures to pesticides. However, farm workers who do not apply pesticide as part of their jobs may still be exposed, and their family members might also be exposed from ‘take-home’ pesticides. People living close to farms are also likely at a high risk of being exposed. Therefore, some epidemiological studies may have underestimated the effect of pesticide exposure when assuming all farmers or pesticide applicators are exposed while non-farmers are un-exposed (non-differential misclassification) [176]. In addition, peak or average exposure intensity may be more relevant than cumulative exposure, especially for characterizing the dose-response relationships [176]. Nevertheless, for asthma, little is known about whether peak exposure has greater relevance than cumulative exposure [23].

### 5.2. Other Issues in the Association Studies

In 1998 pesticide exposure was listed as one of the top respiratory health hazards in agricultural by the American Thoracic Society [183]. Nevertheless, Kirkhorn and Garry in their review of agricultural lung diseases suggested that, although there were notable exceptions, pesticides may not be either a single or direct cause of chronic pulmonary diseases, such as asthma [184]. Other than pesticide exposures, many factors such as variations in genetic makeup, physiological states, socioeconomic and psychosocial factors, and other environmental factors may also contribute to the development of respiratory symptoms and diseases. These factors must be considered along with pesticide exposures when evaluating the effects of pesticide on respiratory health. 

In occupational settings, it is often difficult to identify causes of disease because responses may be delayed and so occur at home, or may even occur many years later for diseases with considerable latency [75]. It is also critical when performing a study to use appropriate ‘normal controls’ to ensure results are compared between comparable groups. In studies of occupational disease, a ‘healthy worker effect’ is often observed, *i.e.*, workers usually have lower rate of disease than the general population [185]. This phenomenon is due to the exclusion of persons with disease from employment [185]. Therefore, the effect of pesticides on respiratory health in occupational settings may be underestimated when comparing with general populations. Le Moual *et al*. showed that a “healthy worker effect” can also occur after employment commences when sick workers leave their jobs perceived as “risky” and find new jobs with less exposures, or become more careful to avoid being exposed within the same job [186]. The author suggested that many cross-sectional studies of occupational asthma lacked sufficient information of health and job status both before and after employment [186]. Spurious results can be obtained without taking account of the ‘healthy worker effect’ into study design, data collection and statistical analyses.

McCauley et al suggested that valid diagnosis or confirmation of symptoms, diseases, or biological markers of a health effect are critical for effectively studying the association between pesticide exposures and health outcomes [187]. Data sources on health outcomes of occupational pesticide exposures include workers’ compensation (WC) systems, hospital and occupational medicine specialist admission and discharge data, and health insurance data. However, workers’ compensation (WC) systems are generally different from region to region, and health insurance information may be incomplete or inaccurate, especially for those part-time workers without health insurance [187,188,189,190]. In addition, longitudinal studies are preferable for characterizing long-term respiratory health effect. Although it is challenging to track cohorts with high mobility, such as migrant and seasonal farmworkers, longitudinal data are especially critical for characterizing the association and establishing a temporal relationship between occupational exposures and health outcome [187,191]. 

In summary, epidemiological studies require careful measurement of both exposures and outcomes when assessing the relationships between occupational pesticide exposures and respiratory health. In addition, proper evaluation of important biases, confounders, and effect modifiers, such as genetic predisposition, social, and psychological factors, are important for avoiding spurious results in association studies. It is also important to use longitudinal approaches when there were temporal variations in pesticide exposures.

## 6. Conclusions

Although this review is not exhaustive in its scope or depth, studies reviewed in this paper have strongly suggested an adverse effect of pesticide exposures on human respiratory health in occupational settings. Respiratory symptoms, including wheezing, airway irritation, dry/sore throat, cough, breathlessness and chest tightness, and respiratory diseases such as asthma and COPD, were associated with occupational pesticide exposures. Impaired lung function was also often observed among people occupationally exposed to pesticides. There is little evidence suggesting that occupational pesticide exposure is associated with respiratory tract infection, although an association has been described for organochlorine insecticide exposures in young children [143,144]. Inconclusive results have been reported from studies of the association between occupational pesticide exposures and lung cancer [104,106,110,111,115,116,117,118]. 

There are some limitations to the data. Although there were studies of populations from developing countries, such as India, Sri Lanka and Ethiopia [48,62,64], most studies have taken place in more developed parts of the world, and many large (and important) areas remain unstudied. In addition, in many studies pesticide exposures were measured by questionnaire-based approaches or something as simple as job title, which has the potential to introduce error. An exception are the studies performed by Sunyer, Boers, Del Prado-Lu, Chakraborty and Karmaus, which used biological measures of pesticide exposure in urine or blood samples [33,48,98,192,193].

Studies have suggested that pesticide management and regulations, educational programs on safety precautions, reinforcement of safety behaviors, especially the proper use of personal protection equipment (PPE) in the workplace, are effective approaches for preventing respiratory symptoms and diseases related to occupational pesticide exposures. 
